# MR imaging features of intracranial primary CNS lymphoma in immune competent patients

**DOI:** 10.1186/1470-7330-14-22

**Published:** 2014-05-07

**Authors:** Asem Mansour, Monther Qandeel, Hikmat Abdel-Razeq, Hussain Ali Abu Ali

**Affiliations:** 1 Department of Diagnostic Radiology, King Hussein Cancer Center, Amman, Jordan; 2 Internal Medicine department, King Hussien Cancer Center, Amman, Jordan

**Keywords:** MR Imaging Features of Intracranial Primary CNS Lymphoma, MRI findings in primary central nervous system lymphoma, PCNSL MRI, DWI in primary central nervous system lymphoma, PCNSL MRI radiology

## Abstract

We aimed to characterize specific MRI findings seen in immune competent patients with intracranial primary CNS lymphoma (PCNSL) and to determine their value in the management of such patients. Pre-treatment MRI examinations of 21 immunocompetent patients with biopsy-proven PCNSL were retrospectively evaluated. T1 and T2 signal characteristics as well as contrast enhancement features are described in all patients. Diffusion, perfusion and proton-MR-spectroscopy features are described in a subset of these patients. In the proper clinical and radiologic setting, suggesting the diagnosis of PCNSL can help institute proper treatment in a timely fashion and avoid unnecessary attempts at surgical resection and the associated morbidity.

## Background

Involvement of the central nervous system by lymphoma can occur in the presence of systemic lymphoma involvement (secondary) or in isolation as a primary CNS neoplasm.

Primary central nervous system lymphoma (PCNSL), the focus of this article, originates in the brain, leptomeninges, spinal cord, or eyes and typically remains confined to the CNS; with only rare spread outside the nervous system. It was initially described by Bailey as perivascular sarcoma [1] and is a rare tumor accounting for 2-6% of all primary brain tumors and 1-2% of all non-Hodgkin lymphomas [2].

PCNSL in the vast majority of patients is a subtype of non-Hodgkin’s lymphoma (NHL), and is usually large cell or immunoblastic type, and is by definition restricted to the CNS (stage IE) [1,3].

PCNSL is encountered in the immunocompetent and in the immunocompromised patients, but the cause and behavior of PCNSL differ based on the affected population. Immunocompromised patients are at particular risk for developing PCNSL which is typically secondary to HIV, organ transplantation, or congenital immunodeficiency syndromes. In this setting, PCNSL arises from Epstein-Barr virus (EBV) infection of B-lymphocytes. In contrast, there is no well-established cause for PCNSL in immunocompetent patient. No correlation has been found between the disease and EBV or the human herpes viruses in this immune competent population [3]. Additionally, it has been and still is a mystery, how these neoplasms develop and grow in the CNS given that B-lymphocytes have no known role in normal brain [3].

There are no pathognomonic imaging features for PCNSL. However, the clinical presentation and imaging features can be quite specific (Table 1). Although brain biopsy remains the gold standard for diagnosis, surgical resection does not improve outcome and chemotherapy remains the mainstay for treatment. Therefore, early diagnosis of CNS lymphoma is crucial for proper management and it is more likely if the correct diagnosis was suggested initially.

**Table 1:** Summary for all MRI findings in 21 patients with primary CNS lymphoma

**Patient**	**Age**	**Sex**	**Location (number of lesions)**	**T1WI**	**T2WI**	**DWI**	**Enhancement**	**Multiplicity**	**Calcifications**	**Hemorrhage**	**Edema**	**Meningeal enhancement**
1	42 y	M	Suprasellar (1)	Iso	Hypo	Restricted	+++	Single	-	-	+++	-
2	32 y	M	HWM (1)	Iso	Iso	Restricted	+++	Single	-	-	+++	-
3	55 y	M	HWM (1)	Hyper	Iso	Restricted	+++	Single	-	+	-	-
4	46 y	F	Cerebellum (1)	Hypo	Iso	Restricted	+++	Single	-	-	++	-
5	65 y	M	HWM (1)	Hypo	Iso	Restricted	+++	Single	-	-	++	-
6	62 y	F	HWM (2)	Hypo	Hyper	Restricted	+++	2 lesions	-	-	++	-
7	68 y	F	HWM (1)	Iso	Hypo	Restricted	+++	Single	-	-	+++	-
8	50 y	M	Subependyma (2), Basal ganglia (1) and Brainstem (1)	Iso	Iso	N\A	+++	4 lesions	-	-	+++	-
9	28 y	M	Basal ganglia (1)	Iso	Iso	Restricted	+	Single	-	-	+++	-
10	39 y	M	HMW (1), Suprasellar (1), Basal ganglia (1)	Iso	Hyper	Restricted	+++	3 lesions	-	-	++	+
11	19 y	M	Cerebellum (2) and HWM (2)	Iso	Iso	Iso	+++	4 lesions	-	-	+++	-
12	45 y	M	Fourth (1) and lateral ventricle (1)	Hyper/Iso*	Iso	Iso	+++	2 lesions	-	+/-*	-	-
13	37 y	M	HWM (2)	Hypo	Hyper	N\A	++	2 lesions	-	-	+	-
14	65 y	M	HWM (3)	Hypo	Iso	Restricted	+++	3 lesions	-	-	+++	+
15	28 D	M	Corpus callosum (Splenium) (1)	Iso	Iso	N\A	+++	Single	-	-	+++	-
16	70 y	F	HWM (1)	Iso	Iso	Restricted	+++	Single	-	-	++	-
17	38 y	F	Basal ganglia (1), Subependyma (1),	Iso	Iso	Restricted	+++	3 lesions	-	-	++	-
			Brain stem (1)									
18	67 y	F	HWM (1)	Iso	Iso	Restricted	+++	Single	-	-	++	-
19	71 y	M	Corpus callosum (Splenium) (1)	Iso	Iso	Iso	+	Single	-	-	-	-
20	37 y	F	HWM (1)	Hypo	Iso	Restricted	+	Single	-	-	++	-
21	47 y	M	Suprasellar (1)	Iso	Hypo	Iso	++	Single	-	-	-	-

HWM: Hemispheric white matter.

N/A: Not available.

*The 4^th^ ventricular lesion is hyperintense (hemorrhagic) and the lesion in the lateral ventricle is isointense.

PCNSL typically involves the supratentorial brain and the lesions are most typically in contact with ventricular or meningeal surfaces. The lesions can also be deeply seated in the parenchyma. The most commonly involved sites include periventricular white-matter, deep gray nuclei, corpus callosum and superficially adjacent to CSF spaces [3]. Atypical locations include brainstem, cranial nerves, cavernous sinuses, pineal gland and pituitary gland.

Hemorrhage or internal calcifications within the tumor are quite rare. This fact contributes to the relatively homogeneous appearance of PCNSL on imaging. When present, hemorrhage and calcification can alter the signal intensity, depending on the age of the hemorrhage and the composition of the calcification [4].

PCNSL usually appears hyperdense on non-contrast enhanced CT scan which reflects hypercellularity of the tumor and the high nuclear/cytoplasmic ratio. This gives on MR imaging the classical iso-to-hypointense signal (relative to grey-matter) on both T1- and T2-weighted images [4-6]. Following intravenous contrast injection, PCNSL tends to show moderate-to-intense enhancement in the majority of cases which implies disruption of the blood–brain barrier. In contrary to the most common primary brain tumor (glioblastoma multiforme), gross necrosis is absent in these tumors. Perilesional edema/signal abnormality is a common feature of PCNSL. It is present in more than 90% of lesions. The edema is mostly moderate-to-severe (larger than the enhancing area) [2].

In this article, we review the specific MR imaging features of PCNSL in immunocompetent individuals which can help radiologists reach the correct diagnosis and start treatment without delay or unnecessary surgical intervention.

## Case presentation

Most of our patients complained from non specific symptoms like headache, seizure and change in mental status. On the other hand, few of them complained from focal neurological deficient typical of a mass effect. Cranial nerve symptoms and vertigo were also noticed in few patients. The systemic symptoms like fever, night sweet and weight loss were very rare.

### Patients and methods

We reviewed the clinical data and imaging features at presentation of 14 male and 7 female immune competent patients with ages ranging from 28 years to 71 years who were diagnosed histopathologically with intracranial PCNSL over a period of 8 years (from January 2004 to August 2012) in King Hussein Cancer Center, Amman, Jordan.

Two experienced radiologists independently reviewed and analyzed the images retrospectively. All scans were reviewed noting lesion location in the brain, size, margin, and the signal characteristics. The presence of calcifications, hemorrhage, perilesional edema, meningeal enhancement and the characteristics of enhancement were also examined. Disagreements were resolved with consensus.

All patients had FLAIR, T2- and T1-weighted images. Post-contrast T1-weighted images were also obtained in all patients. Diffusion-weighted MRI (DW-MRI) was available in 18 patients, while proton-MR-spectroscopy (1H-MRS) was available in 6 patients. Perfusion MR imaging was performed also in 6 patients. Most of the studies were obtained on a 1.5 Tesla Siemens Magnetom Avanto machine. On this machine, the acquired T1 and T2 images were fast spin-echo sequences with TR 500 ms and TE 7.8 ms for T1 and TR 3630 ms and TE 93 ms for T2. For FLAIR, TR 8700 ms and TE 84 ms values were used. Slice thickness was 6 mm for all sequences. T1 sequences were obtained along the three orthogonal planes pre- and post-contrast. The T2 and FLAIR sequences were obtained axially. The DWI was obtained using B-values of 0, 500 and 1000 s/mm2. Two of the later cases were imaged on a 3 Tesla Philips Ingenia machine. The pre- and post-contrast T1 sequences were isotropic, ultrafast spoiled gradient echo sequences (TR 8.3 ms and TE 3.8 ms). For T2, a 5 mm axial fast spin echo sequence with TR 4000 ms and TE 110 ms was obtained. For FLAIR, the TR and TE values were 11000 ms and 125 ms, respectively. MR spectroscopy was performed with a long echo time (135 ms) as a multivoxel 2D exam encompassing the lesion and normal-appearing white-matter.

The contrast used in all these MR exams was OptiMark™ (0.5 mmol/ml gadoversetamide injection from Mallinckrodt Inc.) which was administered intravenously at a dose of 0.2 mL per kg body weight.

T1, T2 and FLAIR sequences were examined qualitatively. Both the DWI images and the ADC maps were examined qualitatively and quantitatively. The mean ADC values in the lesions and in the contralateral normal-appearing white-matter were measured and a ratio was calculated. The MR perfusion maps were also studied in the same way, qualitatively and quantitatively. The cerebral blood volume (CBV) values were determined in the lesions and in the contralateral normal-appearing white-matter and the ratio (rCBV) was calculated. For proton MR Spectroscopy, the ratios of the major metabolites (Choline, N-acetylaspartate and Creatine) were determined. Also, the presence of lipid peaks was determined.

The exams were reviewed on Brilliance Workspace Portal (by Philips). The diffusion and perfusion values were obtained on Syngo Via workstation (by Siemens).

## Results

The 21 patients exhibited 36 lesions. Fourteen of twenty one patients (66.7%) were males. Thirteen patients have a solitary lesion (62%) and the other eight patients have multiple lesions (two lesions seen in three patients, three lesions seen in three patients and four lesions seen in two patients).

**Figure 1:**
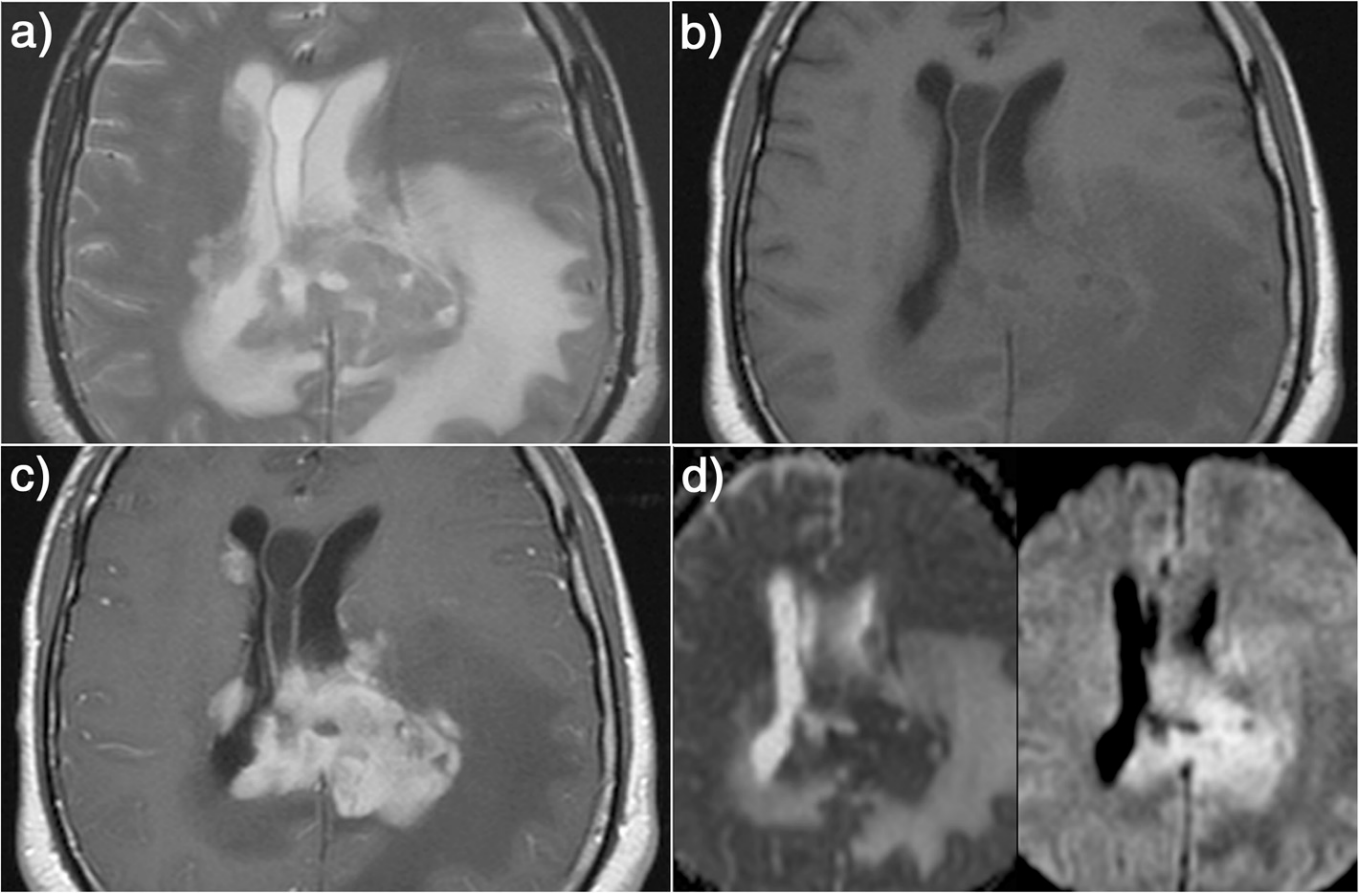
**Axial T2WI, T1WI and contrast-enhanced T1WI as well as ADC and DWI maps show the classic appearance of PCNSL where the mass is isointense to mildly hypointense on T2WI and isointense on T1WI (relative to gray-matter) and shows predominantly solid, intense contrast enhancement (1a-1d).** The mass is also in a typical distribution abutting a CSF-containing space (in the splenium of the corpus callosum abutting the lateral ventricles in this case). The mass is associated with extensive perilesional T2-hyperintensity. On ADC and DWI, the mass shows diffusion restriction.

**Figure 2:**
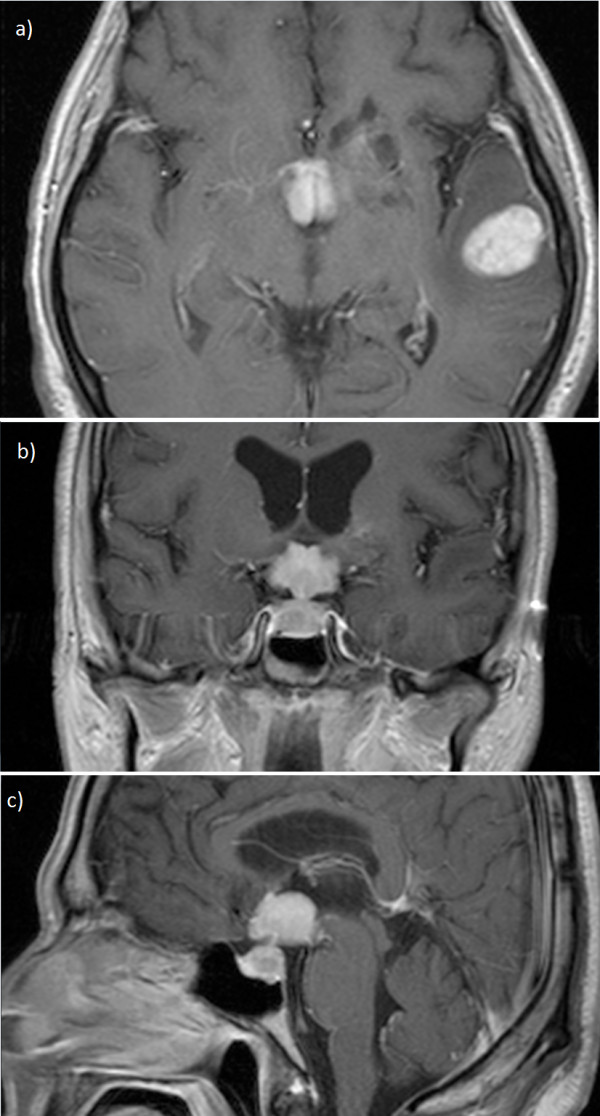
**Post-contrast T1WI in the axial, coronal and sagittal planes show two intensely, solidly contrast-enhancing masses (2a-2c).** The larger one involves the hypothalamus and optic chiasm as well as the pituitary gland. The other one is subcortically located in the left temporal lobe.

Most of the lesions (30 lesions) were located supratentorially (83.3%) and the most common site supratentorially was the hemispheric white-matter (17 lesions (56.7%)). Representative T2WI, precontrast T1WI and postcontrast T1WI, ADC and DWI maps are shown in Figure 1a through 1d. Four lesions were seen in the basal ganglia, 3 lesions in suprasellar region (Figure 2a through 2c), 3 lesions were subependymal, 2 lesions were found in corpus callosum and 1 was in the lateral ventricle. Only 6 lesions were in the infratentorium, three of them in the cerebellum, two in the brainstem and one in the fourth ventricle.

**Figure 3:**
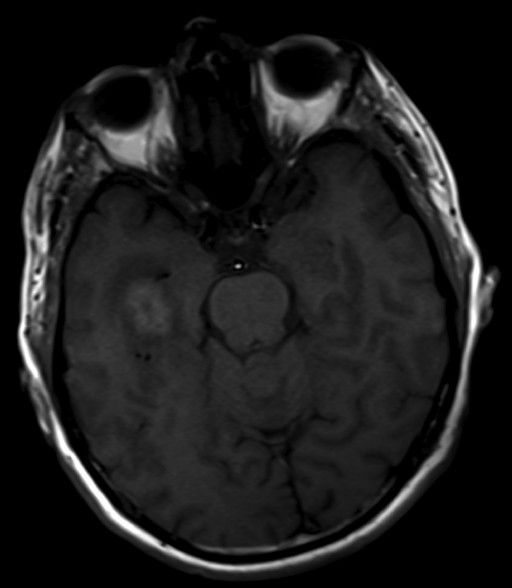
Axial pre-contrast T1WI shows a hyperintense mass in the right temporal lobe.

On T1 sequences, 24 lesions (66.7%) were isointense, 10 lesions (27.8%) were hypointense and only 2 lesions (5.5%) were hyperintense relative to gray-matter (Figure 3). On the other hand, 26 lesions (72.2%) were isointense, 3 lesions were hypointense and 7 lesions (19.5%) were hyperintense on T2 sequences relative to gray-matter.

Perilesional edema or signal abnormality was present in 31 lesions ranging from mild in two lesions, moderate in thirteen lesions and marked in 16 lesions. The degree of edema was judged and reported as follows: a mean diameter of peritumoral edema of less than 10 mm was graded as mild, a mean diameter of 11–20 mm of peritumoral edema was graded as moderate, while marked was used when the mean diameter of peritumoral edema was more than 20 mm. The degree of mass-effect was judged subjectively depending on the degree of displacement and/or compression of the adjacent structures.

**Figure 4:**
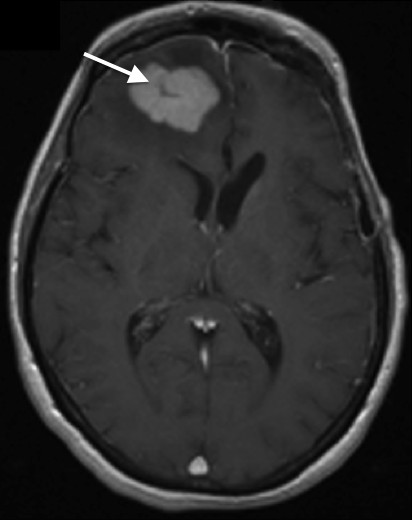
**Axial post-contrast T1WI shows a slit-like center (white arrow) in an otherwise intensely, solidly contrast-enhancing mass in the right frontal white-matter.** The mass causes little mass-effect relative to its large size.

The degree of enhancement was variable. Most of the lesions (30 lesions out of 36) were intensely enhancing (83.3%), three lesions showed minimal enhancement and the remaining three lesions showed moderate enhancement. All lesions were diffusely and homogeneously contrast-enhancing (no necrosis). None of the lesions showed no enhancement. However, some of the lesions showed a slit-like center (Figure 4).

Only two lesions out of 36 showed hemorrhagic changes (6%) and two out of 21 patients showed meningeal enhancement (10%). None of the lesions showed calcification.

Diffusion-weighted MRI (b = 1000 s/mm2) was available in 18 patients. By visual inspection, areas of restricted diffusion were present in 72.4% (n = 21/29) of the lesions in the form of increased signal on DWI and iso to low signal on ADC maps relative to cerebral white-matter (Figure 1d). Mean ADC_lym_/ADC_wm_ for all lesions measured (including the non-diffusion-restricted ones) was 1.4. The ADC ratio range was between 0.91 and 3.00.

**Figure 5:**
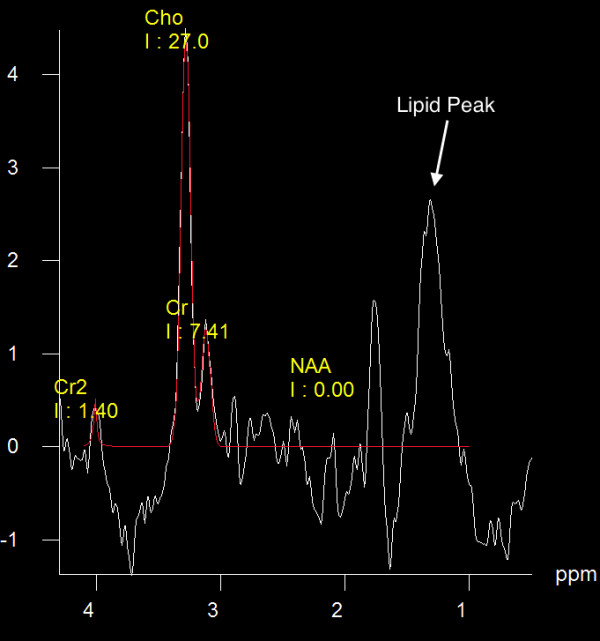
**Spectroscopy at echo time of 135 msec shows elevation of choline (Cho) and marked reduction of N-acetylaspartate (NAA).** In addition, there is a large lipid peak (white arrow).

MR spectroscopy showed marked increase in choline (Cho) and marked reduction in N-acetylaspartate (NAA) peaks (Figure 5). The average Cho/Cr ratio was 3.14 (with a range of 1.67 - 5.82). The average Cho/NAA ratio is 1.2 (with a range of 0.94 – 1.76). There is also a large lipid peak in all cases. This spectroscopic finding was observed in the enhancing component as well as the area adjacent to it (transitional area).

**Figure 6:**
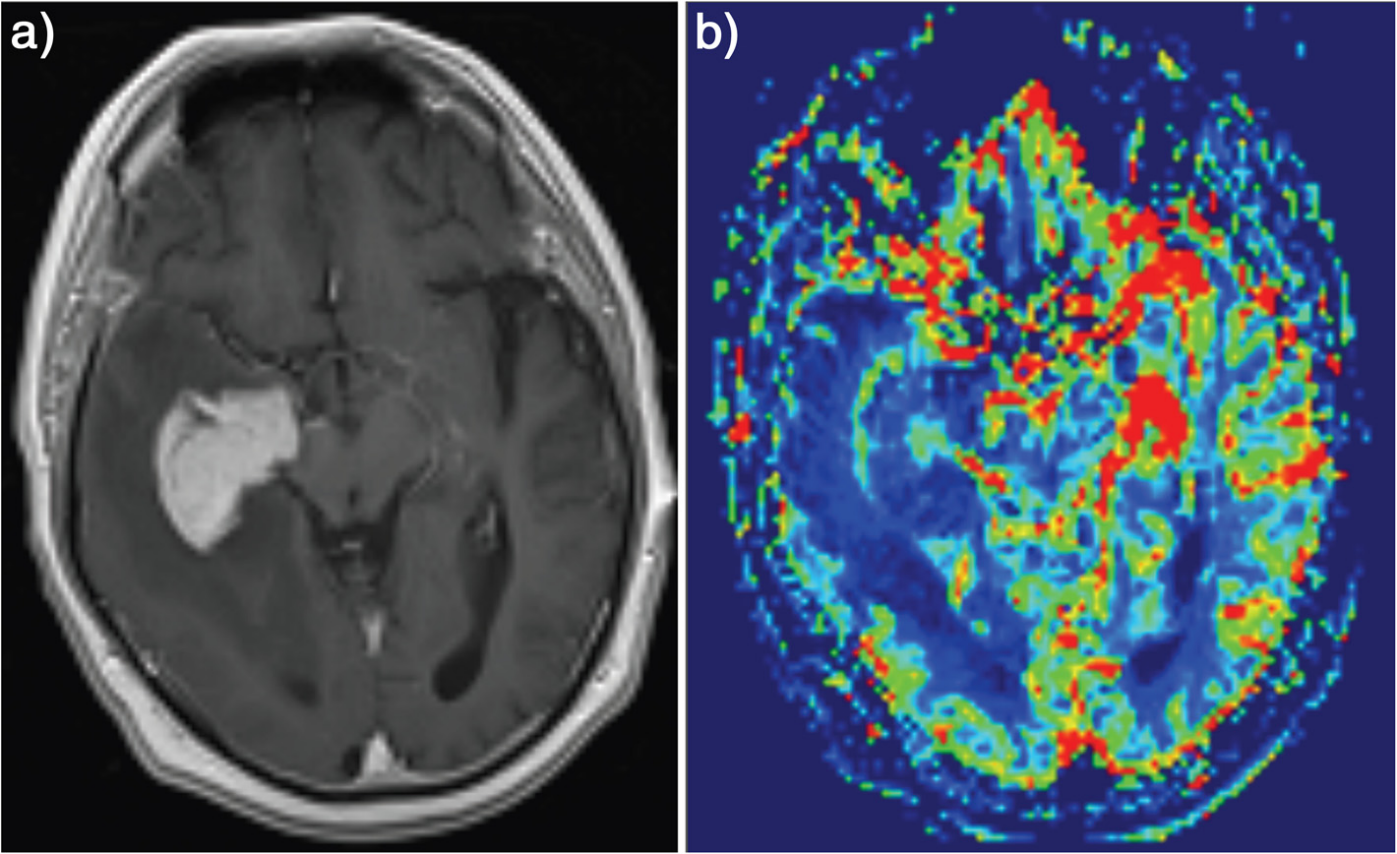
Axial post-contrast T1WI and cerebral blood volume color map from perfusion exam show the reduced cerebral blood volume values in the tumor (6a-b).

Perfusion-weighted MR imaging was performed in 6 patients (total of 8 lesions). PWI shows marked reduction in rCBV (relative to the contralateral normal-appearing white-matter) in 5 lesions (Figure 6a and 6b). The average rCBV was 0.67 (range of 0.03 -2.0).

## Discussion

One of the major issues physicians face while dealing with PCNSL diagnosis, is that it is not a frequent possibility in CNS pathology. In one study by Haldorsen et al. [2], the median time from imaging to diagnosis in non-AIDS PCNSL differed according to the pattern of imaging abnormalities (was 32, 3, 5, and 3 weeks for patients with no, single, multiple, or disseminated lesions, respectively). Patients with no or disseminated lesions were more often diagnosed at postmortem examination [2]. This delay in diagnosis and treatment can negatively impact outcome and prognosis [7].

Although PCNSL has no pathognomonic imaging features, it often has a characteristic appearance on both CT and MR imaging. This is due to multiple factors including its hypercellularity, high nuclear/cytoplasmic ratio, disruption of the blood–brain barrier, and its predilection for the periventricular and superficial regions, often in contact with ventricular or meningeal surfaces [4]. Despite these characteristic imaging features, none of these can unequivocally differentiate CNS lymphoma from other brain lesions [4].

Early diagnosis of PCNSL is especially important given that the treatment of PCNSL differs substantially from other CNS tumors. A visible tumor on imaging is essential to raise the suspicion of CNS lymphoma in the first place. This then can lead to an early histological diagnosis. For this reason steroids should be avoided in suspected PCNSL, with the exception of patients who show evidence of impending brain herniation. A minimally invasive and accurate diagnosis of PCNSL is therefore an important goal that could clearly alter patient treatment and reduce the risk of complication. The preferred method of diagnosis of PCNSL is imaging-guided stereotactic biopsy and CSF cytology [2].

PCNSL is very chemo- and radiosensitive and early diagnosis can significantly affect outcome. Therapeutic advances due to systemic chemotherapy with or without radiation therapy have been reported with median survival times of 33–60 months. Young age and good performance status are favorable prognostic factors [2].

Rarely, PCNSL manifests in a totally different pattern where the brain is diffusely involved. It is called then lymphomatosis cerebri. This distinct entity is typically characterized by diffuse leukoencephalopathy without contrast enhancement, and is considered a diagnostic challenge [8].

### Traditional imaging findings

PCNSL typically presents as a solitary parenchymal mass. Multiple lesions have been reported in 20-40% of non-AIDS PCNSLs. This is in contrast to secondary involvement of the CNS by lymphoma which tends to present with leptomeningeal spread in two-thirds and with parenchymal disease in only one-third of cases [4].

PCNSL typically involves the supratentorial brain and typically the lesions are in contact with ventricular or meningeal surfaces. We observed a similar distribution of the lesions in our population. We also observed few lesions in atypical locations like the brainstem, and cerebellum. Few were suprasellar and intraventricular as well. Hemorrhage or internal calcification within the tumor are atypical features and are quite rare [9]. We observed hemorrhage in only 2 of the 36 lesions which accounted for the two lesions that exhibited T1-hyperintensity in our study. We observed no calcification in any of the lesions. The lack of such atypical imaging features among our patients probably reflects the fact that our patients were all immunocompetent and have not been treated with radiation or antineoplastic agents prior to imaging [10].

PCNSL often has a characteristic appearance on both CT and MR imaging reflecting its hypercellularity and the high nuclear/cytoplasmic ratio. In our patients, we observed isointensity to gray-matter on both T1 and T2 sequences in the majority of lesions (about 70%).

We observed contrast enhancement in all our lesions. It was intense in 30 of the 36 lesions. The contrast enhancement was diffuse and homogeneous in all cases (no ring-like enhancement or necrosis). This is compatible with what has been reported [4]. In the literature, ring-like enhancement was the most common pattern in PCNSL in AIDS patients [4]. Also and in contrary to the most common primary brain tumor (glioblastoma), gross necrosis was absent in our population. Lymphoma has been described to uncommonly present as a non-enhancing white-matter hyperintensity on T2WI (lymphomatosis cerebri). We did not encounter any similar case in our study.

**Figure 7:**
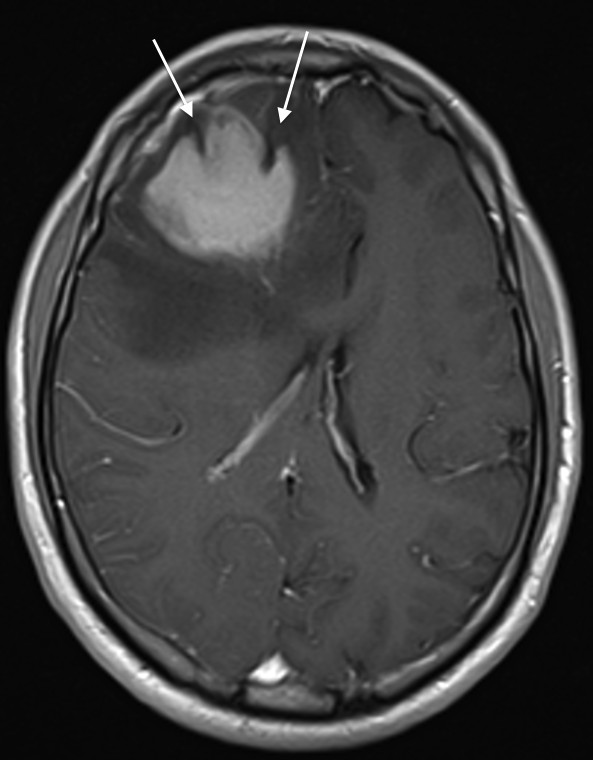
Axial post-contrast T1WI shows a subcortical lesion with two deep indentations reflecting the overlying sulci (two overlapping crescents).

We also noticed that when the PCNSL lesion occurs in subcortical location, it respects the cortex and its morphology (the lesion wraps around the cortex giving the appearance of a crescent) (Figure 7). We observed this in most of the cases located in the subcortical white-matter and it probably reflects the soft, infiltrative nature of these tumors. As far as we know, this is the first description of this feature in association with PCNSL The lesions in our population also did not seem to cause proportionate mass-effect (relative to their size) which can be similarly explained by the soft, infiltrative nature of the disease.

We didn’t encounter any cysts (tumoral or reactive) in our patients. A recent article about brain tumors manifesting as cyst with a mural nodule did cite only one case of PCNSL in which the cysts was related to an adjacent synchronous epidermoid cyst [11,12].

Leptomeningeal enhancement was present in 2 of our 21 patients. The rate of such involvement in our population is slightly higher than what has been reported (1%) [13].

Edema is a common feature of PCNSL. Some published data reports the edema in 77% of the lesions, though usually less prominent than that in malignant gliomas or metastases [4]. We observed edema in 90 of the lesions in our population. The edema was mostly moderate-to-severe, though, as described above, the mass-effect is less than what is expected for the same mass size.

### Diffusion weighted imaging

DWI measures the diffusion of water molecules in biologic tissues. It is a surrogate marker of tumor cellularity. Highly cellular tumors, like CNS lymphoma, would demonstrate a relative decrease in apparent diffusion coefficient (ADC) values [14].

We observed subjective diffusion restriction (hyperintensity on DWI and iso- to hypointensity on ADC maps) in 21 of 29 lesions. Mean normalized ADC values (ADC_lym/_ADC_wm_) for all lesions measured (including the non-diffusion-restricted ones) was 1.4. The ADC ratio range was between 0.91 and 3.00.

These values are slightly higher than what has been described in literature but remain an important clue in differentiating high-grade astrocytoma from lymphomas. In one study, the mean ADC ratio of lymphomas was 1.15 and that of high-grade astrocytomas was 1.68 [15]. In the referenced study, the ADC value also correlated with nuclear-to-cytoplasmic (N/C) ratio on histology.

In addition, pretherapeutic ADC tumor measurement within the contrast-enhancing part of the tumor has been shown to be predictive of the clinical outcome (lower ADC meant shorter progression-free survival and overall survival) [16]. It also can be used as a biomarker to monitor response to treatment, where increasing ADC values suggest favorable response [4].

### Perfusion-weighted MR imaging (MRP)

While contrast enhancement reflects leakiness of the vessels (disruption of the blood–brain barrier), perfusion assesses tumor vascularity. The documented importance of revascularization through angiogenesis for tumor growth has led to a growing interest in such imaging techniques. Perfusion MR and CT imaging visualize nutritive delivery of arterial blood to the capillary bed in the biologic tissue (e.g., tumors). Postprocessing of the acquired data allows for calculation of physiologic parameters, such as cerebral blood volume, cerebral blood flow, mean transit time, and time to peak [4].

In our patient cohort, the average rCBV was 0.84 with a range from 0.23 to 1.9. It was calculated as a ratio of the signal in the lesion relative to the contralateral, normal–appearing white-matter. This is consistent with what has been described in the literature. The low rCBV in lymphoma might be attributed to the characteristic angiocentric growth pattern. This perivascular infiltration also leads to massive leakage of contrast media in the interstitial space which accounts for a characteristic intensity time curve. This low CBV coupled with the characteristic intensity time curve may play an important role in differentiating PCNSL from the other frequently encountered differential diagnosis including glioblastoma multiforme and metastases both of which show significantly higher rCBV values [4].

### MR spectroscopy (MRS)

MRS allows for noninvasive acquisition of biochemical information from biologic tissues. Within a defined volume of interest, signals are registered from chemical nuclei; the most commonly used nuclei are protons (hydrogen).

In our PCNSL patients, MRS (at TE 135 ms) consistently showed increased choline and decreased NAA along with the presence of lipid peak. This is consistent with what has been described in literature [17-19] and while it may not help differentiate PCNSL from glioblastoma multiforme and metastases (which can show similar findings), it may help in differentiating PCNSL from other lesions [4].

We also noticed a transition zone of abnormal spectra outside the enhancing area reflecting the infiltrative pattern of lymphoma which extends beyond the contrast-enhancing region. This might aid in differentiating PCNSL from metastasis but not from high grade glioma.

## Conclusion

PCNSL is a highly malignant tumor which requires different approach in diagnosis and management compared to other high-grade malignant brain tumors. Suggesting the correct diagnosis prior to steroid administration or unnecessary surgical procedures is frequently achievable based on characteristic imaging features. PCNSL typically present as a homogenous mass with T1 and T2 signal intensity that reflects high-cellularity characteristics. The mass typically shows diffuse, homogeneous contrast-enhancement. The lesions typically have less mass-effect than a comparable size metastasis or high-grade glioma. In a subcortical location, the mass may respect the overlying cortex resulting in a crescent-shaped morphology. Necrosis, hemorrhage and calcifications are atypical features and are rare in these tumors.

New imaging techniques (MRS, PWI and DWI) play important role in the diagnosis of PCNSL and differentiating it from other primary and secondary brain tumors (high-grade gliomas and metastases). This is especially important when the characteristic imaging features on traditional imaging are absent.

Comparing what we have found in our population and what has been described in the literature for PCNSL and other brain tumors, we find that PWI can be very helpful in differentiating PCNSL from other high-grade brain tumors which is consistent with the results from other studies. In addition, rCBV ratio is significantly lower in PCNSL compared to what has been described for high-grade glial brain tumors and metastasis. ADC on DWI is also lower in PCNSL compared to the high-grade glial tumors and metastasis, but may not be as accurate as the rCBV in differentiating PCNSL from high-grade glioma (glioblastoma multiforme and anaplastic astrocytoma) and metastases. The lipid peak on MR spectroscopy seems to be a consistent finding as well.

## Consent

Written informed consent was obtained from each patient for the publication of this report and any accompanying images.

## Competing interests

The authors declare that they have no competing interests.

## Authors’ contributions

AM and HA-A collect patients with PCNSL, review the MRI findings and perform the radiological and statistical analysis.MQ drafts the manuscript and perform the radiological and statistical analysis for the DWI part. HA-R follows patients clinically and perform some statistical analysis. All authors read and approved the final manuscript.
